# The PICHFORK (Pain InCHildren Fentanyl OR Ketamine) trial comparing the efficacy of intranasal ketamine and fentanyl in the relief of moderate to severe pain in children with limb injuries: study protocol for a randomized controlled trial

**DOI:** 10.1186/1745-6215-14-208

**Published:** 2013-07-10

**Authors:** Andis Graudins, Robert Meek, Diana Egerton-Warburton, Robert Seith, Trentham Furness, Rose Chapman

**Affiliations:** 1 Monash Health Emergency Medicine Research Group, Department of Medicine, Southern Clinical School, Faculty of Medicine, Nursing and Health Sciences, Monash University, Clayton, VIC, Australia; 2 Pediatric Emergency Department, Monash Medical Center, Monash Health, Clayton, VIC, Australia; 3 Monash Health Emergency Medicine Program, Monash Health, Clayton, VIC, Australia; 4 School of Nursing, Midwifery and Paramedicine, Australian Catholic University, Fitzroy, VIC, Australia; 5 Department of Emergency Medicine, Monash Medical Centre, 264 Clayton Road, Clayton, VIC 3168, Australia

**Keywords:** Intranasal ketamine, Fentanyl, Musculoskeletal injury, Children, Pain, Analgesia

## Abstract

**Background:**

The effectiveness of intranasal (IN) fentanyl as an analgesic for painful pediatric limb injuries in the Emergency Department (ED) has been reported previously. However, efficacy of IN ketamine in sub-dissociative doses is not well studied in the ED setting. A non-blinded pilot study undertaken by this study group suggested that IN ketamine showed similar analgesic effectiveness to that reported with IN fentanyl in similar non-blinded studies. The aim of this randomized, controlled, equivalence trial is to compare the analgesic effect of sub-dissociative dose IN ketamine with IN fentanyl for children with isolated musculoskeletal limb injuries.

**Methods/Design:**

This is a prospective, randomized, controlled, double-blind equivalence trial of children agedthree to thirteen years and less than 50 kg body weight, with isolated musculoskeletal limb injury, presenting to the ED with moderate to severe pain, defined as a verbal response of at least six to a standard 11-point scale (0 = none, 10 = worst pain imaginable). Pain score, sedation, satisfaction with analgesic intervention, and adverse effects will be assessed over a 60 minute interval for each participant. Intranasal ketamine (1 mg/kg) or fentanyl (1.5 microgram/kg) will be administered via blinded syringe and mucosal atomization device in a standardized volume at 0.03 ml/kg, with a maximum volume of 1.5 ml divided equally to both nares. Participants will also receive 10 mg/kg ibuprofen orally. The primary outcome measure will be median change in pain score from pre-administration to 30 minutes post-administration. Non-parametric Mann–Whitney *U* tests will be computed to compare median change in the primary outcome measure for IN ketamine and fentanyl. A sample size of 36 participants per group is needed to detect the expected 40 mm reduction in pain rating with a 95% confidence interval (CI) no greater than ± 10 mm at 30 minutes. Rescue analgesia will be given as IN fentanyl or intravenous morphine.

**Discussion:**

This is the first randomized-controlled trial comparing the efficacy of these two analgesic agents via the intranasal route. If IN ketamine is found to be equally effective to IN fentanyl for this indication, it will provide another analgesic agent that may be considered for the relief of acute pain in children in the ED.

**Trial registration:**

Australian New Zealand Clinical Trials Registry ACTRN12612000795897.

## Background

Children with painful limb injuries frequently present to hospital Emergency Departments (ED) [1]. Accordingly, early and adequate provision of analgesia is considered a fundamental component of quality patient care in the ED [2]. The intranasal (IN) delivery of analgesic agents provides quick and simple drug administration without the need for needles [3], a particular advantage in pediatric patients. Intranasal delivery of fentanyl is a widely accepted pain relief strategy in children both in the pre-hospital [4] and ED [5,6] settings.

Considered the current standard of care in Australian EDs [6], advantages of IN fentanyl can be rapid breakthrough pain treatment [7] comparable to intravenous morphine [8], minimal sedation, and high relative bioavailability [9]. Adverse events specifically attributed to IN fentanyl are difficult to quantify in clinical trials [7], yet patients may be exposed to usual adverse events of opioid-related analgesia. Furthermore, IN fentanyl use is limited due to concentration and dose volume. Intranasal dose volume is, ideally, 0.2 to 0.3 mL per naris, up to a maximum of 0.5 mL [10]. As most EDs only stock the 50 mcg/mL concentration [5], IN fentanyl can only be administered to children up to approximately 50 kg before the introduced volume (0.75 ml) is too large for each naris.

Ketamine is a N-methyl D-aspartate (NMDA) receptor antagonist, in use since the 1960s as an anesthetic, amnesic and analgesic agent. It remains a popular general anesthetic worldwide as it provides sedation without compromising airway reflexes [11]. At sub-dissociative doses, ketamine has been used for pre-operative sedation [12] and analgesia [13] among pediatric patients with little to no reports of adverse events. In a dose-finding pilot study conducted in our tertiary pediatric ED [14] IN ketamine (1 mg/kg) provided analgesia comparable to that reported with IN fentanyl (1.5 mcg/kg) in children with limb injuries [5]. Furthermore, adverse events of IN ketamine were transient and non-severe with dizziness (37%) and poor taste (27%) most commonly reported.

Use of the IN route for the delivery of sub-dissociative doses of ketamine for pain relief is a relatively novel concept in the pediatric population and the analgesic response is not well characterized. To our knowledge, there are no studies comparing the efficacy of IN fentanyl to IN ketamine in children or adults. The primary aim of this clinical trial is to compare the analgesic efficacy of a sub-dissociative dose of IN ketamine with IN fentanyl in pediatric patients presenting to the ED with moderate to severe pain due to isolated musculoskeletal limb injuries. It is hypothesized that IN ketamine and IN fentanyl will provide equally effective pain relief in this patient group.

## Methods

### Design

This is a prospective, randomized, controlled, double-blind equivalence trial. The trial will be conducted in the ED of two Monash Health hospitals: Monash Medical Center, a tertiary referral hospital at which the Pediatric ED has an annual census of 29,000 patients; and Dandenong Hospital, an urban district hospital at which the general ED has an annual census of 57,000 patients, of whom approximately 25% are children. Participant inclusion and exclusion criteria are shown in Table 1.

**Table 1:** Inclusion and exclusion criteria of the ‘PICHFORK’ trial

**Inclusion criteria**	**Exclusion criteria**
Children aged three to thirteen years and up to 50 kg body mass	Inability to gain informed consent from parent or guardian
Children with an isolated musculoskeletal injury of the limb	Treatment with serotonergic antidepressants, prior administration of parenteral or IN analgesics or opioid analgesia, and opioid antagonist use
Reported pain severity greater than or equal to 6 in the standard 11-point verbal rating scale (0 = none, 10 = worst pain imaginable) at triage	Children with allergy to ketamine, fentanyl, or ibuprofen
Children that would normally be considered for IN fentanyl administration for analgesia at Monash Health EDs.	Aberrant nasal anatomy, acute or chronic nasal problems or nasal trauma that may preclude adequate intranasal delivery
	Presence of multiple trauma and/or head injury with loss of consciousness, and/or cognitive impairment.

Note: Use of simple analgesia such as paracetamol or ibuprofen or inhalational methoxyflurance within four hours prior to ED arrival does not exclude participation. *ED* emergency department, *IN* intranasal.

### Randomization and storage

Forty numbered syringes will be block-randomized at each site to contain either ketamine or fentanyl using a four block-randomization table as described by Altman and Bland (1999) [15]. Each syringe will be prepared as allocated and the unique number will be recorded on the patient prescription accompanying the syringe.

### Study medications

Fentanyl citrate 50 mcg/ml (DBL Fentanyl, Hospira Pty Ltd, Melbourne, Australia) and Ketamine 200 mg/ml (Ketalar^®^ Hospira Pty Ltd, Melbourne, Australia). For both fentanyl and ketamine, 3 ml syringes will be prepared. Fentanyl syringes will be made by drawing up 1.5 mL of fentanyl citrate parenteral solution for injection (50 mcg/mL) into a 3 mL syringe. Ketamine syringes will be prepared by diluting 0.5 mL ketamine parenteral solution (100 mg/mL) with 1.0 mL of sodium chloride 0.9% to make a final concentration of 50 mg/1.5 mL (33.3 mg/mL). The trial syringes will be given an expiry of one week from manufacture. Four syringes of the block-randomized batch will be prepared weekly by the pharmacy at each hospital site. Each week, unused syringes will be deemed expired and destroyed by the pharmacy. Unused, expired syringes will be re-made with the same numbers and drug allocations until all syringe numbers are accounted for. Syringe production rate may vary through the study period depending on the recruitment rate.

### Measurement tools

#### ***Pain severity rating scales***

Pain rating will be undertaken using a similar methodology to that used in our pilot study [14]. The Faces Pain Scale - Revised (FPS-R): for children aged three to six years, requires choice of one of a line of six faces described as progressing from ‘no pain’ at the left hand end, to ‘very much pain’ at the right hand end. The faces are allocated numerical ratings of 0, 20, 40, 60, 80 and 100 from left to right. This rating is used for severity calculations at a single time point, while change is recorded as positive for face selection towards the left (less pain) and negative towards the right (more pain).

Visual Analog Scale (VAS): for children aged seven years and older, required marking of a standard 100 mm line labeled ‘no pain’ at the left hand end and ‘worst pain ever’ at the right hand end. The severity rating at a single time point is the measurement (mm) from the left hand end of the line. Change in severity is recorded as positive for movement to the left (less pain) and negative towards the right (more pain).

#### ***Description of change in pain severity***

The patient will be asked to describe change in pain severity as; ‘a lot less’, ‘a little less’, ‘the same’, ‘a little more’ or ‘a lot more’.

#### ***Patient/parent/guardian satisfaction of pain change***

The patient/parent/guardian will be asked to describe satisfactions of pain severity change as; ‘satisfied’, ‘not satisfied’ or ‘no opinion’.

#### ***Sedation***

The description of sedation will incorporate assessment by the medical staff member and the patient/parent/guardian. The attending doctor will assess the degree of sedation using the University of Michigan Sedation Scale (UMSS). This has been validated previously in analgesic and sedation studies in children [16]. The patient/parent/guardian will be asked to subjectively describe the degree of sedation in their child as; ‘too sedated’, ‘sedated enough’, ‘unchanged’ or ‘not sedated enough’.

### Procedure

On identification of an eligible patient (Table 1), the attending doctor will read a standardized verbal consent script to the parent/guardian. On obtaining verbal consent, baseline severity ratings and sedation score will be recorded. The study medication will be obtained from the next numbered study pack and administered intranasally using a mucosal atomizer device (MAD^®^ Wolf Troy Medical, Salt Lake City, UT, USA). For analgesic dose-volumes equal to and less than 0.5 mL, the entire dose will be administered in one nare. Doses above 0.5 mL will be divided equally and administered to both nares. Oral ibuprofen (10 mg/kg) (FENPAED AFT Pharmaceuticals Pty Ltd., Sydney, Australia) will then be administered within the first 15 minutes of IN administration of the study medication unless this drug has already been given in the past four hours. The parent/guardian will then be given the full written participant information to procure written consent for ongoing participation and data collection. Review of need for additional analgesia will occur across the 60-minute data collection period. If rescue analgesia is needed, participation in the study will be terminated at that time. Data collected to time of termination will be retained and included in the statistical analyses. Rescue analgesia will be either further IN fentanyl or intravenous morphine depending upon the treating doctor’s preference.

### Outcome measures

The primary outcome measure will be median reduction in pain severity scores 30 minutes (T30) after study medication administration. Secondary outcome measures will include; VAS change in pain severity 15 and 60 minutes (T15, T60), subjective description of change in pain severity, satisfaction of pain change, sedation, adverse events, need for rescue analgesia at T15, T30 and T60, and need for rescue analgesia at T15, T30 and T60.

### Statistical analyses

Patient age, sex, and weight will be reported as median with interquartile range or number and percentage and compared using either Mann–Whitney *U* or Fisher’s exact tests as appropriate. Pain severity at each time point and change in severity between time points will be reported as median millimeter (mm) with interquartile range and compared using the Mann–Whitney *U* test. Previous research supports combination of severity measures from the VAS and FPS-R [16]. The UMSS scores will be reported as median with interquartile range and compared using Mann–Whitney *U* test. Patient/parent satisfaction and need for rescue medication will be reported as number and percentage and compared using Fisher’s exact test. Adverse events will be described as type and incidence.

### Sample size

The required sample size of 36 per group has been calculated using the assumption that the desired primary clinical outcome is that pain should be at least ‘a little’ better by 30 minutes post-study medication administration. Based on previous reports of response to analgesia in children [14,17] we defined a successful response to analgesia in this study as a reduction of VAS pain score measurement of approximately 40 mm from baseline with a 95% confidence interval (CI) no greater than 10. To allow for some margin of error in the underlying assumptions, the baseline sample size has been increased by 10% to 42 patients per group.

## Discussion

### Scope of the trial

While a number of descriptive observational studies have reported the analgesic effects of IN ketamine in sub-dissociative doses in non-ED settings in adults, no trials have compared the efficacy of ketamine to other analgesic agents in a blinded fashion. There is a paucity of literature describing the use of IN ketamine in acute pain in the ED in either adults or children. As a result, this study aims to assess the effectiveness of IN ketamine and fentanyl in a systematic blinded fashion in the ED.

### Strengths

Prior to our dose-finding study [14], the effectiveness of IN ketamine in an ED population was unreported, with inferences drawn from other populations. However, results of our initial un-blinded study in children with limb injuries suggested that IN ketamine has the potential to be an effective analgesic agent in acute pain in children. Confirmation of these observations in a blinded and randomized fashion will provide a higher level of evidence as to the potential efficacy of IN ketamine.

### Limitations

This trial may be limited by selection bias as some participants with eligible limb injuries may have already received opioid analgesia prior to ED presentation. As a result, this will be a non-consecutive, convenience data set. Furthermore, we will not exclude children who have received non-opioid oral analgesia in this study. Patients with a pain score greater than 6/10 on presentation can be assumed to have a failed response to non-opioid analgesia and as a result, would normally require opioid analgesia for pain relief. However, it is likely that some patients receiving non-opioid analgesia prior to presentation may have a reduction in pain scores below those that would allow recruitment in this study. This also has the potential to prolong the recruitment period for our target sample size.

### Ethical considerations

This clinical trial has the Human Research Ethics Committee (HREC) approval of Monash Health HREC Committee B. The trial is registered with the Australian New Zealand Clinical Trials Registry (ACTRN12612000795897). The trial protocol has been reviewed by the Monash Health Drug and Therapeutics committee. A trial safety monitoring committee has been incorporated into the study design to review adverse event incidents and ongoing analgesic efficacy in the trial.

### Summary

Provision of IN analgesia is a common practice for pediatric patients in the ED. Currently, IN fentanyl is the medication of choice based upon data form observational studies. We aim to assess whether the efficacy of IN ketamine is comparable to that of IN fentanyl in children with moderate to severe pain and isolated limb injuries. A positive result in this study may result in considering the addition of ketamine as an alternative analgesic to IN opioids in children with moderate to severe pain in the ED setting.

## Trial status

Recruitment commenced in November 2012 for the 84 participants needed for the trial. It is anticipated recruitment will be completed by the end of 2013.

## Abbreviations

CI: Confidence interval; ED: Emergency department; FPS-R: Faces Pain Scale - Revised; IN: Intranasal; NMDA: N-methyl D-aspartate; UMSS: University of Michigan Sedation Scale; VAS: Visual Analogue Pain Score.

## Competing interests

The authors declare they have no competing interests.

## Authors’ contributions

AG, the principle investigator, conceived, designed, and submitted the study for ethics committee assessment and approval. RM was responsible for statistical design and sample size computation. DE-W, BS, TF, and RC each made substantial contribution to one or more of: study design, preparation of the manuscript and critical revision. All authors read and approved the final manuscript.
